# In vivo functional significance of direct physical interaction between Period and Cryptochrome in mammalian circadian rhythm generation

**DOI:** 10.1093/pnasnexus/pgae516

**Published:** 2024-11-15

**Authors:** Junko Kawabe, Kohhei Kajihara, Yohei Matsuyama, Yukiya Mori, Teruki Hamano, Mai Mimaki, Yukari Kitamura, Ritsuko Matsumura, Makoto Matsuyama, Masahiro Sato, Masato Ohtsuka, Koichi Node, Makoto Akashi

**Affiliations:** The Research Institute for Time Studies, Yamaguchi University, Yamaguchi 753-8511, Japan; The Research Institute for Time Studies, Yamaguchi University, Yamaguchi 753-8511, Japan; The Research Institute for Time Studies, Yamaguchi University, Yamaguchi 753-8511, Japan; The Research Institute for Time Studies, Yamaguchi University, Yamaguchi 753-8511, Japan; The Research Institute for Time Studies, Yamaguchi University, Yamaguchi 753-8511, Japan; The Research Institute for Time Studies, Yamaguchi University, Yamaguchi 753-8511, Japan; The Research Institute for Time Studies, Yamaguchi University, Yamaguchi 753-8511, Japan; The Research Institute for Time Studies, Yamaguchi University, Yamaguchi 753-8511, Japan; Division of Molecular Genetics, Shigei Medical Research Institute, Okayama 701-0202, Japan; Department of Genome Medicine, National Center for Child Health and Development, Tokyo 157-8535, Japan; The Institute of Medical Sciences, Tokai University, Kanagawa 259-1193, Japan; Department of Molecular Life Science, Division of Basic Medical Science and Molecular Medicine, Tokai University School of Medicine, Kanagawa 259-1193, Japan; Department of Cardiovascular Medicine, Saga University, Saga 849-8501, Japan; The Research Institute for Time Studies, Yamaguchi University, Yamaguchi 753-8511, Japan

## Abstract

In the current model, the auto-negative feedback action of Period (Per) and Cryptochrome (Cry) on their own transcription is the hallmark mechanism driving cell-autonomous circadian rhythms. Although this model likely makes sense even if Per and Cry undertake this action in a mutually independent manner, many studies have suggested the functional significance of direct physical interaction between Per and Cry. However, even though the interaction is a biochemical process that pertains to the fundamentals of the circadian oscillator, its in vivo contribution to circadian rhythm generation remains undefined. To answer this question, we focused on zinc coordination between Per and Cry, whose contribution to circadian rhythm generation remains undefined. Specifically, we aimed to impair endogenous Per–Cry association by introducing an amino acid substitution to zinc-coordinating residues located at the Per1 and Per2 C-terminal facing Cry in mice. These mice did not show severe impairment in the Per–Cry physical interaction, but rather a shortened period and decreased robustness in circadian rhythms at the tissue-autonomous and whole-body levels. Furthermore, these mice also showed a decrease in Per half-life, suggesting that impaired fine-tuning of Per half-life caused abnormal circadian period and robustness in vivo. We also found a minor but significant impact of a reindeer-specific Per2 mutation located in the Per–Cry interface on circadian rhythms in vivo. These lines of evidence indicate that only partial impairment of the Per–Cry physical interaction produces a substantial effect on circadian period and robustness, supporting the in vivo functional significance of the interaction.

**Significance Statement**

The current model of the mammalian circadian clock indicates that the auto-negative feedback action of Per and Cry on their own transcription is the hallmark mechanism in circadian rhythm generation. While this model likely makes sense if these two proteins undertake this action independently, many studies suggest the significance of the direct physical interaction between these proteins. To reveal the in vivo significance of the interaction, we focused on a zinc ion located at the Per–Cry interface and a reindeer-specific mutation located on the interface and found that the direct physical interaction between endogenous Per and Cry controls the half-life of Per and affects the period and robustness of circadian rhythms.

## Introduction

Most organisms exhibit diurnal rhythms in physiology and behavior, which are driven by the circadian clock (1, 2). The internal oscillator enables gene expression at appropriate times of the day, allowing organisms to anticipate and adapt to the earth's rotation. The circadian clock is entrained in response to environmental cues such as light. The suprachiasmatic nucleus (SCN), the central clock located above the optic chiasma in the hypothalamus, orchestrates peripheral clocks located in almost all cells over the whole body (3–6). The circadian clock is cell-autonomous and consists of auto-negative feedback loops of transcription (7–11). In the mammalian core negative feedback loop, the BMAL1 and CLOCK transcription factor complex activates the *Period* (*Per*) and *Cryptochrome* (*Cry*) genes via E-box enhancer elements (12, 13). Subsequently, the transcription repressor Cry, together with Per, suppresses the BMAL1 and CLOCK complex (14–16). This repetitive negative feedback-driven transcriptional oscillation with a period of ∼24 h thus drives circadian rhythms (17–20). Although a redox system can be a cell-autonomous circadian oscillator independently of transcription (21), this oscillator may be dispensable for circadian rhythm generation in vivo (22). The traditional core negative feedback loop of transcription therefore remains considered the major in vivo circadian oscillator.

The molecular model described above strongly indicates that the auto-negative feedback action of Per and Cry on their own transcription is the hallmark process in the realization of cell-autonomous circadian rhythm generation. Although this model likely works even if Per and Cry play this role in an independent manner, it has been considered that the direct physical interaction between Per and Cry controls their own intracellular dynamics, consequently contributing to the robustness and modulation of the molecular oscillator. Indeed, numerous in vitro studies have suggested the functional significance of the Per–Cry interaction; specifically, the control of their intracellular localization and half-life has been reported to be the most significant factor of the interaction (23–26). Given these previous in vitro studies, it is intuitively plausible that the Per–Cry physical interaction plays a pivotal role also in vivo. However, because the role of the physical interaction in circadian rhythms has not been investigated in vivo, the possibility cannot be excluded that the interaction is dispensable for circadian rhythm generation. Conventional gene knockout approaches cannot answer this question.

An understanding of the in vivo significance of the physical interaction between endogenous Per and Cry requires examination of whether impairment of the interaction affects circadian rhythms at the tissue-autonomous and whole-body levels. To do this, we first focused on a zinc ion located at the Per–Cry interface whose presence was revealed by determination of the crystal structure of the Per2–Cry1 or Per2–Cry2 complex (27, 28). It was shown that the zinc ion may be involved in redox-dependent control of the Per–Cry interaction. Interestingly, a previous study reported that mice carrying a transgene coding Cry1^C414A^ protein harboring a single amino acid substitution in its zinc interface showed a longer free-running circadian period in comparison with control mice (29–31), suggesting the possibility that zinc coordination is required for normal circadian rhythm. However, experimental results from transgenic mice are generally difficult to interpret because the genomic location and copy number of a transgene are unclear and its expression levels are excess and constant. In the case of *Cry1^C414A^* mice, the situation was further complicated because these mice carried not only this transgene but also intact endogenous *Cry* genes. The in vivo contribution of zinc coordination to circadian rhythm generation therefore remains unknown. In addition to zinc coordination, we also focused on a reindeer-specific spontaneous mutation which may be responsible for the severe loss of circadian rhythm (32). Although this mutation is located on the Per2 interface facing Cry, the question of whether it really impairs circadian rhythms has not been investigated.

In this study, we aimed to reveal the in vivo functional significance of the direct physical interaction between Per and Cry using mice carrying mutated zinc-coordinating residues of Per1 and/or Per2 and mice carrying an amino acid substitution corresponding to the reindeer-specific mutation of Per2.

## Results

### Effect of amino acid substitution of the zinc-coordinating residues on the binding intensity and intracellular localization of Per and Cry

**Fig. 1.**
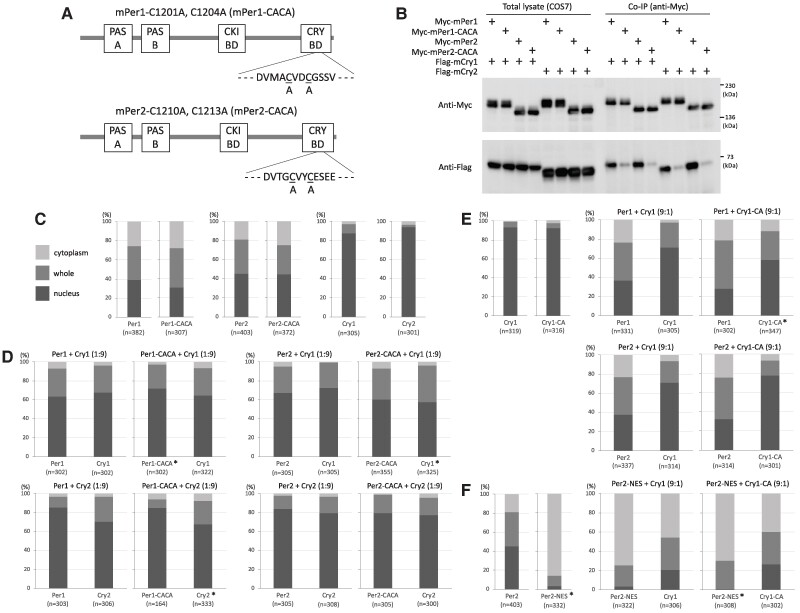
Effect of amino acid substitution of zinc-coordinating residues on the binding intensity and intracellular localization of Per and Cry. A) Schematic representation of expression constructs for mouse Per1 and Per2 whose both two zinc-coordinating residues located in the C-terminal Cry-binding domain are substituted from cysteine to alanine. PAS, Per–Arnt–Sim domain; CKI-BD, casein kinase I-binding domain; CRY-BD, Cry-binding domain. B) COS7 cells were transfected with expression vectors in the indicated combinations, and whole-cell extracts were subjected to immunoprecipitation using anti-MYC antibody. Western blotting was performed with the indicated antibodies. Size marker bands are indicated on the right. COS7 cells were transfected with a single (C) or two (D to F) expression vectors in the indicated combinations. Ratio in parentheses represents the amount ratio of the Per and Cry expression vectors. The intracellular localization of Myc-tagged Per and Flag-tagged Cry was detected by immunostaining transfectants. Their intracellular localization was classified into three categories: cytoplasm, light gray; whole, gray; and nucleus, dark gray, respectively. Percentages of these three categories are shown as a bar graph. The number of cells examined is shown in parentheses under each bar graph. Experiments were performed twice with similar results. *P < 0.01 (Fisher's exact test, control vs mutant).

Before introducing amino acid substitution to the zinc-coordinating residues in mice, we examined whether the coordination was necessary for the physical interaction between Per1 and Cry, given that previous studies have shown its importance in that between Per2 and Cry (27, 28). Expression vectors for Per1 and Per2 whose zinc-coordinating residues were substituted from cysteine to alanine were constructed and transfected with a Cry expression vector into COS7 cells (Fig. 1A). Immunoprecipitation experiments were performed using cells transfected in the indicated combinations between Per1 or Per2 and Cry1 or Cry2 expression vectors. The results clearly demonstrated that zinc coordination was necessary for the physical interaction between Per1 and Cry in a manner similar to Per2 (Fig. 1B). Next, we investigated the effect of the decrease in binding intensity on intracellular localization of Per and Cry by immunostaining transfectants. Amino acid substitution of the Per zinc-coordinating residues did not affect the intracellular localization of Per itself (Fig. 1C). An excess amount of Cry over Per induced an increase in nuclear Per, and this intracellular distribution was closely similar between wild-type Per and Per^CACA^ proteins (Fig. 1D). In contrast, Per induced an increase in cytoplasmic Cry independently of the ratio of Per and Cry (Fig. 1D and E), and this intracellular distribution was almost the same between wild-type Cry and Cry^CA^ proteins, whose zinc-coordinating residue was substituted from cysteine to alanine (Fig. 1E). When Per2 was ectopically fused with a strong nuclear export signal to induce a drastic change in intracellular localization of Cry from nucleus to cytoplasm, the intracellular distribution between wild-type Cry and Cry^CA^ proteins again remained closely similar (Fig. 1F).

### Effect of amino acid substitution of zinc-coordinating residues on circadian rhythms in mouse locomotor activity

**Fig. 2.**
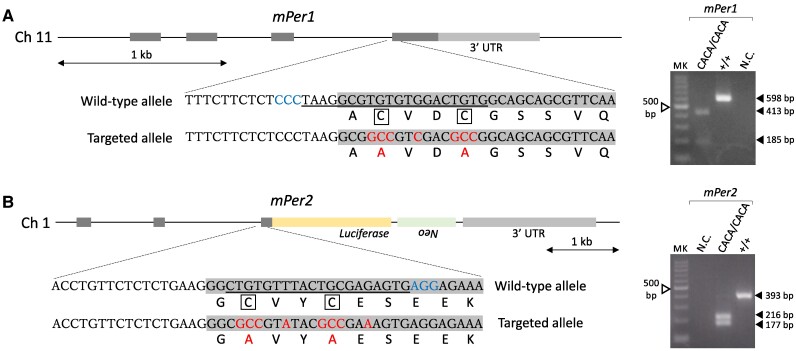
Generation of mice carrying amino acid substitutions in zinc-coordinating residues of Per1 or Per2. (A and B, Left) Genomic structures of the *mPer1* and *mPer2* genes and coding and amino acid sequences including zinc-coordinating residues (enclosed cysteines). *Per2^Luc^* knock-in mice were used for mutagenesis. To introduce the same amino acid substitution to Per1 and Per2 as shown in Fig. 1A, a genome editing method called i-GONAD was performed. Protospacer adjacent motif (PAM) sequences, crisprRNA targets, and resulting mutations are indicated by blue, underline, and red, respectively. (A and B, Right) Examples of PCR-based genotyping. Homozygotes carrying the double amino acid substitutions in the zinc-coordinating residues of Per1 or Per2 were obtained by mating heterozygotes. Before performing agarose gel electrophoresis, PCR products including the wild-type or targeted sequence were digested using a specific restriction enzyme; amplicons derived from the targeted sequence of *Per1^CACA/CACA^* or *Per2^CACA/CACA^* mice were recognized by *Sal*I or *Bst*1107I, respectively. MK, 100-bp ladder marker; NC, negative control.

The results of the intracellular localization assay suggest that Per–Cry binding affinity remains at least partially intact under an intracellular environment. Although we therefore estimated that severe loss of Per–Cry binding affinity required substitution of multiple amino acids or deletion of a wide range of amino acids because the Per interface with Cry widely extends to its C terminus (27, 28, 33), these kinds of modification may cause severe errors in the C-terminal structure of Per. The present amino acid substitution approach therefore has an advantage regarding this technical concern, although the induced decrease in Per–Cry binding intensity may be limited. *Per2^Luc^* knock-in mice were used for this mutagenesis to evaluate tissue-autonomous circadian rhythms with high accuracy. To introduce the same amino acid substitution to Per1 and Per2 as shown in Fig. 1A, we performed a method called improved-Genome editing via Oviductal Nucleic Acids Delivery (i-GONAD) (34–36). Briefly, surgical procedures were performed on anesthetized females at day 0.7 of pregnancy. The oviduct was exposed after making an incision at the dorsal skin, and a genome editing cocktail was injected into the oviduct lumen using a micropipette. Immediately after the injection, the oviduct regions were grasped with electrodes and in situ electroporation was performed. These procedures were conducted for *Per1* or *Per2* separately. In both the *Per1* and *Per2* target cases, one of seven females gave birth to three F0 mice. The F0 generation was subjected to PCR-based amplification of the targeted genomic region and subsequent restriction enzyme digestion of the PCR products to identify candidate mice harboring an expectedly edited genome. In both the *Per1* and *Per2* target cases, all F0 mice showed an expectedly sized amplicon, indicating that the indel was not large, and one of three F0 mice potentially carried an expectedly targeted allele. Genomic sequencing of the PCR product from this F0 mouse to confirm the success of genome editing revealed that its nucleotide sequence was completely identical to our designed sequence (Fig. 2A and B, left). This F0 mouse carrying a *Per1^CACA^* or *Per2^CACA^* allele was mated with wild-type *Per2^Luc^* mice to confirm the transfer of the edited genomic region to the next generation. Finally, homozygotes were obtained by mating heterozygotes (Fig. 2A and B, right) and used for subsequent experiments.

**Fig. 3.**
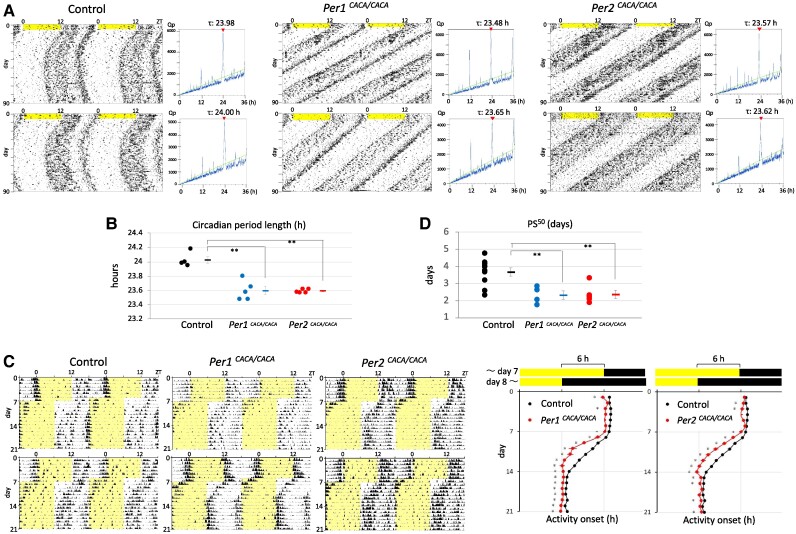
Effect of amino acid substitution of zinc-coordinating residues on circadian rhythms in mouse locomotor activity. A) Representative double-plotted actograms from control, *Per1^CACA/CACA^* and *Per2^CACA/CACA^* mice. Two mice from each genotype are shown. Locomotor activity was monitored using an infrared sensor under constant darkness, after habituation to the environment inside a dark box (12-h light and 12-h dark). Light period is shaded in yellow. Periodograms are shown beside each actogram. Green line indicates Qp values at *P* = 0.05. Values above periodograms are the estimated period length. B) Free-running period length in control, *Per1^CACA/CACA^* and *Per2^CACA/CACA^* mice (24.03 ± 0.04 [SEM] h, *n* = 4; 23.60 ± 0.06 [SEM] h, *n* = 5; 23.59 ± 0.01 [SEM] h, *n* = 5, respectively). Each circle and square indicate individual and average free-running period length, respectively. **P < 0.01 (two-tailed t test, control vs mutant). (C, Left) Entrainment to 6-h advanced light and dark (LD) cycles was monitored using an infrared sensor. Two mice from each genotype are shown. Light period is shaded in yellow. (C, Right) Daily activity onset (mean ± SEM) in the 6-h advanced LD cycles was plotted (control, *n* = 10; *Per1^CACA/CACA^*, *n* = 4; *Per2^CACA/CACA^*, *n* = 5). *P < 0.05 (two-tailed t test, control vs mutant). D) 50% PS^50^ calculated using datasets in C. Each circle and square indicate individual and average PS^50^, respectively. Error bars indicate SEM. **P < 0.01 (two-tailed t test, control vs mutant).

To evaluate the effect of these amino acid substitutions on circadian rhythms at the whole-body level, circadian rhythms in free-running locomotor activity were monitored under constant darkness after habituation to regular 12-h light and 12-h dark cycles and the environment inside the dark box. The day-by-day advance of activity onset in actograms clearly indicates a shortened circadian period length in both *Per1^CACA/CACA^* and *Per2^CACA/CACA^* mice, and this shorter period was confirmed by a periodogram analysis (Fig. 3A). The period length of circadian rhythms in both CACA mice was significantly shorter than that of control mice (Fig. 3B). Resynchronization of free-running rhythms to rescheduled light and dark cycles likely becomes more delayed in mice carrying a more robust oscillator. We therefore expected that these CACA mice may show an accelerated resynchronization due to decreased circadian robustness caused by reduced Per–Cry binding intensity. Both mutant mice entrained to 6-h advanced light and dark cycles significantly faster than control mice (Fig. 3C). Consistent with this, the 50% phase shift value (PS^50^) showed a statistically significant difference between the control and mutant mice (Fig. 3D).

### Effect of amino acid substitution of zinc-coordinating residues on tissue-autonomous circadian clocks

**Fig. 4.**
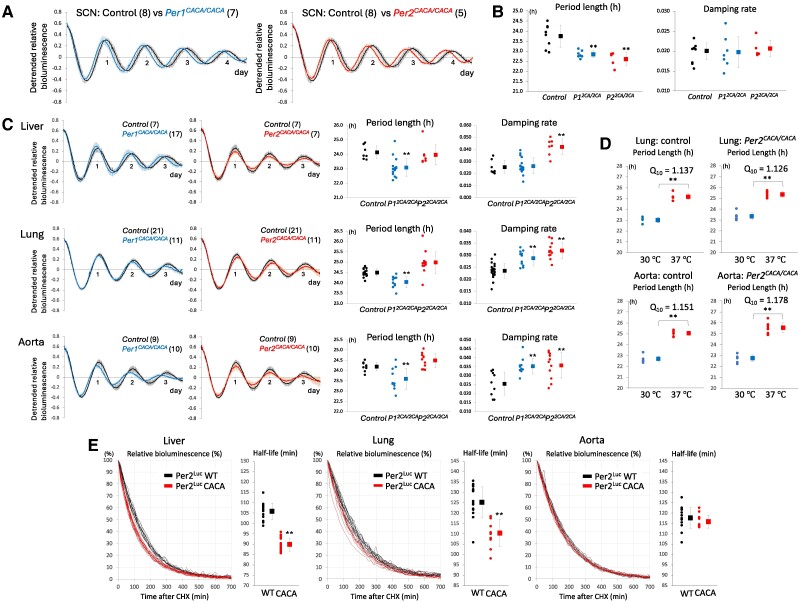
Effect of amino acid substitution of zinc-coordinating residues on tissue-autonomous circadian clocks. A) Circadian rhythms in SCN slices from control, *Per1^CACA/CACA^* and *Per2^CACA/CACA^* mice were detected by real-time PER2^Luc^ bioluminescence monitoring. The detrended datasets were obtained by subtracting the 24-h simple moving average from the raw data. The first amplitude was set to 1. The number of replicates examined is shown in parentheses. Graphs show the mean of replicates ± SD. B) Period length and damping rate of circadian rhythm in SCN slices in each genotype calculated from datasets represented in A. The number of slices used was eight in control, seven in *Per1^CACA/CACA^*, and five in *Per2^CACA/CACA^* mice. Each circle and square indicate individual and average value, respectively. Error bars indicate SD. **P < 0.01 (two-tailed t test, control vs mutant). (C, Left) Circadian rhythms in liver, lung, and aorta slices from each genotype were detected by real-time PER2^Luc^ bioluminescence monitoring. The datasets were detrended, and the first amplitude was set to 1. The number of replicates examined is shown in parentheses. Graphs show the mean of replicates ± SD. (C, Right) Period length and damping rate in these slices. Each circle and square indicate individual and average value with SD. **P < 0.01 (two-tailed t test, control vs mutant). D) Lung and aorta slices were cultured at the different temperatures of 30 and 37 ℃, and temperature coefficient Q_10_ was calculated for circadian period length. Each circle and square indicate individual and average period length with SD. **P < 0.01 (two-tailed t test, 30 vs 37 ℃). (E, curve graph) Time-dependent decrease in Per2^Luc^-derived bioluminescence emitted from ex vivo cultured liver, lung, and aorta was detected after administration of CHX at the first circadian peak. (E, dot graph) Per2^Luc^ half-life was calculated by fitting a model curve to raw degradation curve. Each circle and square indicate individual and average Per2^Luc^ half-life with SD. **P < 0.01 (two-tailed t test, control vs mutant).

To examine the effect of amino acid substitution of zinc-coordinating residues on tissue-autonomous circadian clocks, in addition to the central clock SCN, tissue explants from the liver, lung, and aorta were cultured ex vivo. Circadian rhythms of bioluminescence emitted from SCN explants of both *Per1^CACA/CACA^* and *Per2^CACA/CACA^* mice showed no significant difference in damping rate in comparison with control mice but a significantly shorter period than control mice (Fig. 4A and B). As for peripheral clocks, *Per1^CACA/CACA^* mice showed a significantly shorter circadian period in all of the liver, lung, and aorta explants and a significant increase in damping rate in lung and aorta explants, in comparison with control mice (Fig. 4C). In contrast, all of these peripheral clocks in *Per2^CACA/CACA^* mice showed no significant difference in circadian period length but a significant increase in damping rate, in comparison with control mice. Lung and aorta explants were cultured at the different temperatures of 30 and 37 ℃, and temperature coefficient Q_10_ was calculated for circadian period length (Fig. 4D). Temperature compensation in peripheral clocks was not affected by the CACA mutation.

Next, to investigate the effect of amino acid substitution of the Per2 zinc-coordinating residues on the Per2 half-life, the time-dependent decrease in Per2^Luc^-derived bioluminescence emitted from ex vivo cultured liver, lung, and aorta was detected in real time after administration of cycloheximide (CHX) at the first circadian peak (Fig. 4E). The data clearly showed a faster decrease in bioluminescence in ex vivo cultured liver and lung of *Per2^CACA/CACA^* mice than those of control mice. A half-life estimation confirmed that Per2 degradation was significantly faster in these tissues of *Per2^CACA/CACA^* mice than those of control mice.

### Effect of simultaneous introduction of amino acid substitution to Per1 and Per2 zinc-coordinating residues on circadian rhythms in vivo

**Fig. 5.**
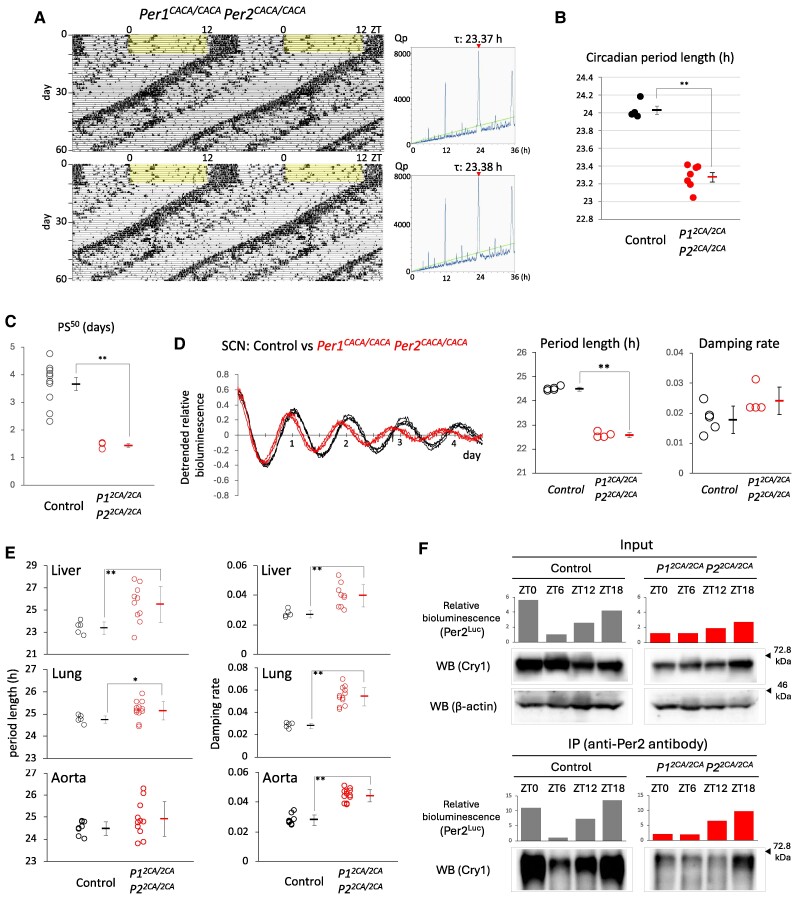
Effect of simultaneous introduction of amino acid substitution to Per1 and Per2 zinc-coordinating residues on circadian rhythms in vivo. A) Representative double-plotted actograms and periodograms of double CACA mutant mice. After habituation, mice were subjected to constant darkness. The green line in the periodograms indicates Qp at *P* = 0.05. Values above periodograms are period length. B) Each circle and square indicate individual and average period length of free-running activity, respectively. **P < 0.01 (two-tailed t test). The period length is 24.03 ± 0.04 (SEM) h in control (*n* = 4) and 23.27 ± 0.05 (SEM) h in double mutant (*n* = 7). C) Each circle and square indicate individual and average PS^50^, respectively. Error bars indicate SEM. *P < 0.05, **P < 0.01 (two-tailed t test). (D, Left) Circadian rhythms in SCN slices were detected by real-time bioluminescence monitoring. The detrended datasets are shown. The first amplitude was set to 1. (D, Middle and Right) Period length and damping rate of circadian rhythm in SCN slices in each genotype. The number of slices used was five in control and four in double mutant mice. Each circle and square indicate individual and average value, respectively. Error bars indicate SD. *P < 0.05, **P < 0.01 (two-tailed t test). E) Circadian rhythms in liver, lung, and aorta slices from each genotype were detected by real-time bioluminescence monitoring, and period length and damping rate were calculated. Each circle and square indicate individual and average value with SD, respectively. *P < 0.05, **P < 0.01 (two-tailed t test). F) The liver was harvested at the indicated time points, and homogenates were subjected to immunoprecipitation using anti-Per2 antibody. The relative amount of Per2^Luc^ protein was detected as bioluminescence. For detection of Cry1 and β-actin proteins, western blotting was performed with the indicated antibodies. Arrowheads indicate the position of size markers.

Given gene redundancy, we generated double CACA mutant mice carrying both the *Per1^CACA^* and *Per2^CACA^* alleles in a double homozygous manner. Circadian rhythms in free-running locomotor activity showed a ∼0.7-h shorter period length in these double mutant mice than that in control mice (Fig. 5A and B). This average period length was shorter than that for both *Per1^CACA^* and *Per2^CACA^* single homozygous mice shown in Fig. 3B. Double CACA mutant mice entrained to 6-h advanced light and dark cycles faster than control and single CACA mutant mice, and the PS^50^ values showed a statistically significant difference between control and double CACA mutant mice (Fig. 5C). Circadian rhythms in Per2^Luc^ bioluminescence obtained from ex vivo cultured SCN showed a ∼2-h shorter period length in double CACA mutant mice than that in control mice, and this period length was also shorter than that for both *Per1^CACA^* and *Per2^CACA^* single homozygous mice shown in Fig. 4B (Fig. 5D). However, circadian oscillation remained robust without detectable damping. In contrast to the central clock, circadian rhythms in three peripheral tissues of double CACA mutant mice showed a more variable and longer period length than those of control mice due to severe damping (Fig. 5E).

Next, we examined whether zinc coordination was necessary for the physical interaction between endogenous Per and Cry proteins, as shown using overexpressed proteins in Fig. 1. Co-immunoprecipitation of endogenous Per2 and Cry1 proteins was performed using liver extracts harvested at zeitgeber time (ZT) 0, 6, 12, and 18 (Fig. 5F). We were not able to detect Per2^Luc^ by western blotting even after repeated optimization of experimental conditions, probably because this fusion protein was oversized. We therefore detected a relative amount of the fusion protein as bioluminescence intensity with high quantitative performance. The result from input samples containing a nearly uniform amount of liver tissue indicated that the CACA mutation caused a moderate decrease in the total amount of endogenous Per2 and Cry1 proteins, probably due to their destabilization caused by a decrease in their binding intensity. Unexpectedly, unlike overexpression experiments in Fig. 1, co-immunoprecipitation of endogenous Per2^CACA^ and Cry1 proteins revealed an impaired but obviously detectable physical interaction between these proteins in the liver of double CACA mutant mice.

### Effect of introduction of a reindeer-specific spontaneous mutation to the corresponding residue of mouse Per2 on circadian rhythms

**Fig. 6.**
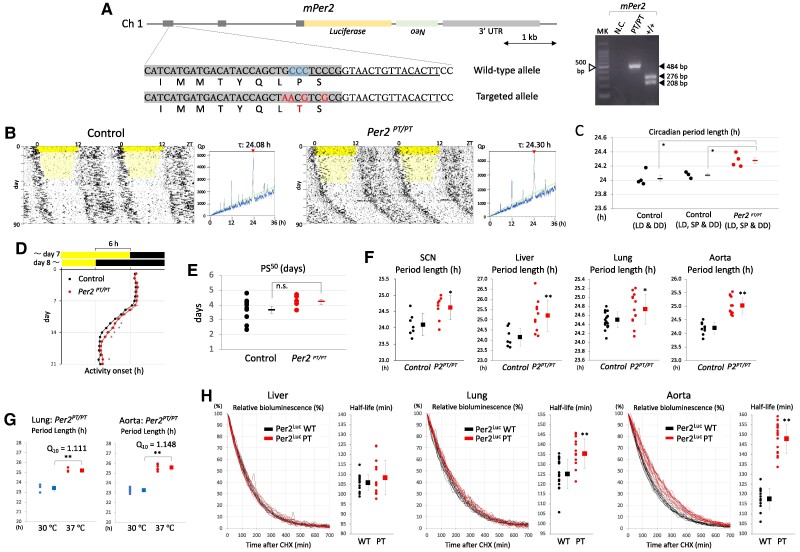
Effect of introduction of a reindeer-specific spontaneous mutation to the corresponding residue of mouse Per2 on circadian rhythms. (A, Left) Genomic structure and amino acid sequences including a residue corresponding to the reindeer-specific mutation P1172T. i-GONAD was performed using *Per2^Luc^* mice. Protospacer adjacent motif sequences, blue; crisprRNA targets, underline; resulting mutations, red. (A, Right) Examples of PCR-based genotyping. Amplicons derived from control mice were recognized by *Pvu*II. MK, 100-bp ladder marker; NC, negative control. B) Representative double-plotted actograms and periodograms. Prior to constant darkness, mice were subjected to a gradual short photoperiod (SP) (<10 lux, faint yellow). Green line indicates Qp at *P* = 0.05. Values above periodograms are period length. C) Each circle and square indicate individual and average period length. *P < 0.05 (two-tailed t test, control vs mutant). Left control mice were not exposed to a gradual SP. Free-running period length is 24.03 ± 0.04 (SEM) h, *n* = 4; 24.08 ± 0.02 (SEM) h, *n* = 3; 24.28 ± 0.04 (SEM) h, *n* = 4, from the left. D) Entrainment to 6-h advanced LD cycles. Daily activity onset (mean ± SEM) was plotted (control, *n* = 10; *Per2^PT/PT^*, *n* = 5). *P < 0.05 (two-tailed t test, control vs mutant). E) PS^50^ calculated using datasets in D. Each circle and square indicate individual and average PS^50^ with SEM. ns, not significant (two-tailed t test). F) Circadian rhythms of Per2^Luc^ bioluminescence in tissue slices. Each circle and square indicate individual and average circadian period length with SD. *P < 0.05, **P < 0.01 (two-tailed t test, control vs mutant). G) Lung and aorta slices were cultured at 30 or 37 ℃, and temperature coefficient *Q*_10_ was calculated for period length. Each circle and square indicate individual and average period length with SD. **P < 0.01 (two-tailed t test, 30 vs 37 ℃). (H, curve graph) Time-dependent decrease in Per2^Luc^-derived bioluminescence from ex vivo cultured slices, after administration of CHX at the first circadian peak. (H, dot graph) Per2^Luc^ half-life was calculated by fitting a model curve to raw degradation curve. Each circle and square indicate individual and average half-life with SD. **P < 0.01 (two-tailed t test, control vs mutant).

To further investigate the in vivo functional significance of the Per–Cry physical interaction, we generated additional mutant *Per2^Luc^* mice carrying the Per2 P1156T mutation corresponding to the reindeer-specific spontaneous Per2 P1172T mutation (Fig. 6A) (32). As in the case of CACA mice, genome editing was performed using the i-GONAD method. Although control and *Per2^PT/PT^* mice were subjected to a gradual short photoperiod condition (<10 lux) imitating the arctic light environment prior to constant darkness, there was no obvious difference in circadian locomotor activity between genotypes. However, both actograms and periodograms indicate that *Per2^PT/PT^* mice showed a significantly longer period in the circadian rhythms of locomotor activity under constant darkness (Fig. 6B and C). *Per2^PT/PT^* mice showed a slightly slower entrainment to 6-h advanced light and dark cycles in comparison with control mice (Fig. 6D), although there was no significant difference in PS^50^ among the genotypes (Fig. 6E). Ex vivo tissue culture was performed to evaluate the effect of the PT mutation on tissue-autonomous circadian oscillators. Consistent with circadian phenotypes in locomotor activity, the central and peripheral clocks of PT mice showed a significantly longer circadian period length (Fig. 6F). According to the values of temperature coefficient Q_10_ for circadian period length, temperature compensation in peripheral clocks was not affected by the PT mutation (Fig. 6G). To examine the effect of the PT mutation on Per2 half-life in ex vivo cultured tissues, a time-dependent decrease in Per2^Luc^-derived bioluminescence was monitored after administration of CHX at the first circadian peak (Fig. 6H). The data clearly showed a slower decrease in bioluminescence in ex vivo cultured lung and aorta of *Per2^PT/PT^* mice than those of control mice. A half-life estimation confirmed that Per2 degradation was significantly slower in these tissues of *Per2^PT/PT^* mice than control mice.

## Discussion

### Effect of artificial amino acid substitution of zinc-coordinating residues on circadian rhythms in mice

To impair the in vivo association between endogenous Per and Cry, we applied amino acid substitution to the zinc-coordinating residues of Per proteins in mice. While previous in vitro studies have examined zinc coordination of Per2 with Cry proteins, we additionally targeted the zinc-coordinating residues of Per1. More specifically, we generated mice carrying the double amino acid substitution C1201A and C1204A in Per1 (*Per1^CACA/CACA^* mice) or C1210A and C1213A in Per2 (*Per2^CACA/CACA^* mice) using a genome editing method. Consistent with previous in vitro experiments using recombinant Per2 (27, 28), our immunoprecipitation experiments using transfectants confirmed that the physical interaction in all four combinations between Per1 or Per2 and Cry1 or Cry2 required zinc coordination. Importantly, the CACA mutation in Per1 or Per2 resulted in a ∼25-min shorter circadian period length in locomotor activity under constant darkness, in comparison with control mice. Given that Per1 and Per2 are functionally overlapping (37, 38), the relatively mild circadian phenotypes in these mutant mice are not unexpected. Fast entrainment to phase-advanced light and dark cycles in these mutant mice may suggest a decreased robustness of their circadian clock. Thus, these behavioral analyses of CACA mice revealed that zinc coordination at the interface between endogenous Per and Cry has functional significance in circadian rhythm generation at the whole-body level, and in a more generalized interpretation, the biochemical process of direct physical interaction between endogenous Per and Cry is necessary for robust circadian rhythm generation in vivo.

### Mechanism of the negative effects of artificial amino acid substitution of the zinc-coordinating residues of Per on circadian rhythms in vivo

In many cases, circadian characteristics at the whole-body level reflect those at the cell-autonomous level (39, 40). We therefore hypothesized that circadian phenotypes in CACA mutant mice as described above were attributable to circadian abnormalities in the cell- or tissue-autonomous circadian oscillator. As expected, the central clock SCN of *Per1^CACA/CACA^* and *Per2^CACA/CACA^* mice cultured under an ex vivo condition showed a ∼1-h shorter circadian period length in comparison with control mice. As for peripheral clocks under an ex vivo condition, while all tissues examined consistently showed a shorter circadian period length in *Per1^CACA/CACA^* mice than in control mice, the change in circadian period length was not statistically significant in all tissues of *Per2^CACA/CACA^* mice. This unexpected result in *Per2^CACA/CACA^* mice may be because of decreased detection accuracy of circadian period change due to a decreased circadian robustness or an increased damping rate of circadian rhythms at the tissue-autonomous level. Together, as hypothesized, circadian phenotypes in CACA mice may be attributable to circadian abnormalities in tissue-autonomous circadian clocks.

Previous in vitro experiments have shown that the major role of the direct physical interaction between Per and Cry is the control of their own intracellular localization and half-life (23–26). As described above, while immunoprecipitation experiments confirmed that the CACA mutation caused a severe defect in the binding intensity of Per and Cry, co-transfection-based intracellular localization assays unexpectedly demonstrated that their localization was nearly intact even in the absence of zinc coordination. This may be inconclusive at this time because this assay was performed using overexpressed artificial proteins with much higher intracellular concentrations than endogenous proteins. However, even allowing for the possibility of potential artifacts, because the intracellular distribution of Per and Cry was closely similar in the presence and absence of zinc coordination, these results strongly suggest that zinc coordination may be dispensable for their mutually dependent and normal intracellular localization. In contrast, ex vivo experiments revealed that zinc coordination was clearly involved in the control of Per half-life, although a tissue-specific difference was identified. Together, the decrease in Per half-life in CACA mice likely at least partially causes a shortened period length and decreased robustness in tissue-autonomous circadian clocks, subsequently resulting in circadian phenotypes at the whole-body level.

Per1 and Per2 proteins are functionally redundant (37, 38). We therefore performed phenotypic analyses using double CACA mutant mice, *Per1^CACA/CACA^ Per2^CACA/CACA^* mice. Circadian rhythms in not only free-running activity but also Per2^Luc^ bioluminescence from ex vivo cultured SCN showed a shorter period length in double CACA mutant mice than both single CACA mutant mice. Additionally, circadian rhythms in three peripheral tissues of double CACA mutant mice also showed a variable period length, probably due to severe damping. However, although these results indicated that zinc coordination was necessary for normal and healthy circadian rhythms, circadian oscillation remained robust at the central clock and whole-body levels. This conclusion is basically the same as that from single CACA mutant mice. Unlike the results from overexpressed proteins in Fig. 1, co-immunoprecipitation of endogenous Per2^CACA^ and Cry1 proteins unexpectedly revealed an impaired but detectable physical interaction. This explains why circadian oscillation remained robust even in double CACA mutant mice. Given that double mutant mice showed a ∼0.7-h shorter period in free-running activity, a ∼2-h shorter period in the SCN, and severely damped and unstable oscillation in peripheral clocks even though the effect of the CACA mutation on the Per–Cry physical interaction was limited in vivo, these results indirectly indicate a potentially large contribution of the interaction to circadian rhythm generation in vivo.

Our hypothesis explaining the difference in co-immunoprecipitation experiments between overexpressed and endogenous proteins is as follows. Our transfection-based intracellular localization experiment showed that the CACA mutation had little effect on the Per–Cry physical interaction, indicating that this mutation did not severely impair their binding intensity in an intracellular environment. The mutation-induced impairment in the physical interaction between overexpressed Per and Cry proteins was detectable only in an artificial buffer environment. In contrast, the lack of any severe dissociation between endogenous Per2^CACA^ and Cry1 in an artificial buffer environment was probably because some endogenous factors compensated for the mutation-induced moderate impairment of binding intensity. The amount of these endogenous compensatory factors is expected to be too small to protect the binding between overexpressed Per–Cry proteins. Taking these ideas together, we speculate that CACA mutation does not severely impair binding intensity between Per and Cry proteins and that some endogenous factors strongly but not completely compensate for this impaired binding in vivo.

### Effect of the introduction of a reindeer-specific spontaneous Per2 mutation to the corresponding residue on mouse circadian rhythms

We revealed the in vivo functional significance of the Per–Cry physical interaction using CACA mice, as mentioned above. We also aimed to investigate the significance of this interaction using other mutant mice (*Per2^PT/PT^* mice) carrying the Per2 P1156T mutation, corresponding to the reindeer-specific spontaneous Per2 P1172T mutation (32). Reindeer distributed around arctic regions have no clear circadian rhythms (41, 42), and this mutation is considered potentially responsible for the phenotype. Because this mutation is located on the Per–Cry interface, and because the in vitro binding intensity between Per2 and Cry was severely impaired by introducing this mutation to the corresponding residue (32), this abnormality in Per2–Cry binding intensity may potentially at least partially contribute to the severe loss of circadian rhythms. However, the question of whether this mutation really negatively affects circadian rhythms in vivo remains unanswered.

Importantly, we found that PT mice showed a significantly longer circadian period length in locomotor activity than control mice. Although we were concerned about the possibility that the development of circadian defects in mice may require simultaneous introduction of other reindeer mutations of Per2 in addition to the PT mutation (32), this result demonstrates that the PT mutation is sufficient to induce a minor but significant circadian abnormality in mice. This finding in PT mice supports the in vivo functional significance of the Per–Cry direct physical interaction shown above using CACA mice. Unexpectedly, however, although functional defects in the circadian clock likely result in accelerated entrainment to rescheduled light and dark cycles, the PT mutation did not cause accelerated entrainment to these cycles, but rather slightly delayed entrainment. With regard to tissue-autonomous circadian clocks of PT mice, the central and peripheral clocks of PT mice showed a significantly longer circadian period length than those of control mice. Again unexpectedly, in contrast to the CA mutations, the PT mutation resulted in a longer Per2 half-life, albeit in a tissue-dependent manner. Taken together, these findings indicate that the increase in Per2 half-life in PT mice likely at least partially causes a longer period length of circadian rhythms at the tissue-autonomous and whole-body levels. Together, given these results from both the CACA and PT mutations, circadian period length at the tissue-autonomous and whole-body levels could be determined in a manner proportional to the Per half-life length.

We speculate about why the PT mutation unexpectedly caused Per2 stabilization and lengthened circadian period length, whereas the CACA mutation expectedly caused Per2 destabilization and shortened circadian period length due to an impaired Per–Cry physical interaction, as follows. Previous studies reporting a crystal structure of the Per2–Cry complex showed that the proline was located between two α helices of the Per2 C-terminal facing Cry (27, 28). The proline is highly conserved but not in direct contact with Cry. Accordingly, the PT mutation might not directly affect the Per2–Cry physical interaction, which in turn means that the mechanism by which the PT mutation impairs circadian rhythms is likely different to that in the CACA mutation. Consistent with this hypothesis, unlike the CACA mutation, previous findings on co-immunoprecipitation using overexpressed proteins revealed that simultaneous introduction of multiple amino acid substitutions to Per2 residues which included this proline did not affect the Per2–Cry1 physical interaction (28). Confusingly, however, the paper which originally reported the reindeer-specific mutation showed that the PT mutation caused a decrease in Per2–Cry1 binding intensity using a similar transfection-based co-immunoprecipitation method (32). This discrepancy may be dependent on differences in experimental conditions and suggests that the impairment of binding intensity may not be severe. As we show in the present study using both overexpressed and endogenous Per2^CACA^ proteins, nonsevere impairment of binding intensity is likely protected through endogenous compensatory factors. It should be noted that proline interferes with the formation of protein secondary structures because its N terminus forms a five-atom ring with the side chain. This property supports the hypothesis that the PT mutation results in facilitation or stabilization of neighboring secondary structure formation, which leads to delayed turnover of the Per2–Cry complex due to the stabilization of Per2 and consequently to longer circadian rhythms in physiology and behavior.

## Conclusions

Given that only partial impairment of the physical interaction between endogenous Per and Cry proteins caused obviously detectable defects in circadian period and robustness at the tissue-autonomous and whole-body levels, the direct physical interaction between these proteins conclusively contributes to normal circadian period and robustness in vivo. More specifically, the present study demonstrates that the in vivo physical interaction between endogenous Per and Cry controls the half-life of endogenous Per and that this fine-tuning of half-life might be required for the normal period and robustness of circadian rhythms at the tissue-autonomous and whole-body levels. However, whether the interaction is indispensable to circadian rhythm generation remains unclear. Along with these main findings, the present study demonstrates for the first time the in vivo relevance of Per amino acid residues previously identified to be potentially important for Per functions, specifically residues that coordinate zinc on the Per–Cry interface and a residue whose mutation is potentially responsible for circadian dysfunction in arctic reindeers.

### Limitations of the study

There are several limitations and questions for future study. First, although the results from the PT mutation support the in vivo functional significance of the Per–Cry physical interaction, circadian phenotypes in PT mice seem to be inconsistent with severe loss of reindeer circadian rhythms. Given that there are two spontaneous mutations in reindeer Per2 in addition to P1172T, simultaneous introduction of multiple amino acid substitutions to these corresponding residues in mice may answer this question. Second, our intracellular localization assay excluded the functional contribution of zinc coordination to mutually dependent intracellular localization of Per and Cry. A high performance method to detect the intracellular distribution of endogenous Per and Cry is required to verify this result. Finally, although only the Per half-life was examined in the present experiments, circadian phenotypes in CACA and PT mice might also be dependent on the Cry half-life. Given previous studies showing that Per and Cry affect the stability of each other (23–26), we speculate that the Cry half-life likely changes in a manner responsive to the Per half-life. Verification of this hypothesis requires an experimental tool to detect the half-life of endogenous Cry with high sensitivity, a tool similar to the *Per2^Luc^* mice used in the present study.

## Materials and methods

### Expression constructs, cell culture, and transfection

Expression constructs for mutants of mPer1 and mPer2 were generated by amplifying the entire insert and plasmid vector (pcDNA3, Invitrogen) with inverse PCR using back-to-back primers containing designed mutations, and linear PCR products were subsequently self-ligated to reform the circular plasmid vector. The entire sequence of the coding sequence for mPer1 and mPer2 was verified in each construct to confirm the presence of designed mutations and the absence of unexpected PCR errors. COS7 cells were grown in Dulbecco's modified Eagle’s medium (DMEM) supplemented with antibiotics and 10% fetal bovine serum (FBS), and cultured in 5% CO_2_. The total amount of DNA was adjusted to 2 mg with empty pcDNA3, and transfection was performed with Lipofectamine 3000 (Invitrogen) according to the manufacturer's instructions.

### Immunoprecipitation

For immunoprecipitation experiments, COS7 cells transfected with expression vectors were lysed and the livers harvested from euthanized mice were homogenized in an immunoprecipitation buffer (33). These lysates were then subjected to centrifugation at 15,000 rpm for 15 min, and the supernatants were incubated with antibody-bound protein G-Sepharose beads (Pharmacia) at 4 °C for 2 h. After the incubation, the immunoprecipitants were washed with fresh buffer, treated with Laemmli sample buffer, and subjected to western blot analyses. The sources of antibodies for immunoprecipitation and western blotting are as follows: anti-Flag antibody (M2) was from Sigma, anti-Myc antibody (9E10) and anti-Per2 antibody (H90) were from SantaCruz, and anti-Cry1 and anti-β-actin antibodies were from Medical & Biological Laboratories (PM081 and PM053, respectively).

### Immunocytochemistry

To detect intracellular localization of Per and Cry, immunocytochemistry was performed as described previously (33). The experimental procedure is briefly as follows. COS7 cells were cultured on a cover glass in 6-well plates and transfected with the indicated combination of vectors using Lipofectamine 3000. Approximately 24-h after transfection, the cells were fixed with 3.7% formaldehyde in phosphate buffered saline and permeabilized with Triton X-100. After blocking with bovine serum albumin, cells were incubated with anti-Flag (M2, Sigma) and anti-Myc (A14, SantaCruz) antibodies, followed by antimouse IgG conjugated with Alexa 488 (Thermo Fisher Scientific) and antirabbit IgG conjugated with Alexa 594 (Thermo Fisher Scientific). The cells were mounted with Vectashield mounting medium (Vector Laboratories), and the intracellular localization of Per and Cry was visually determined under a fluorescence microscopy.

### Animals

*Period2::luciferase* (*Per2^Luc^*) knock-in C57BL6 mice were provided from Dr Joseph Takahashi (43). All mice were bred and maintained on regular 12-h light and dark (LD) cycles and allowed ad libitum access to food and water. The number and age of mice used in each experiment are indicated in the corresponding figure legends. All protocols for animal experiments were approved by the Animal Research Committee of Yamaguchi University. Animal studies were performed in compliance with the Yamaguchi University Animal Care and Use guidelines.

### Preparation of CRISPR–Cas9 electroporation solution

To perform in vivo mutagenesis by genome editing, the synthetic crRNA and tracrRNA were commercially purchased as Alt-R CRISPR guide RNAs together with Alt-R S.p. Cas9 Nuclease 3NLS from Integrated DNA Technologies. For knock-in-based mutagenesis, the single-stranded oligodeoxynucleotide (ssODN) donors were custom synthesized by Eurofins Genomics. The sequence of crRNAs and ssODNs used in this study is listed in Table S1. To reconstitute each nucleic acid, ssODNs were suspended in RNase-free water to a concentration of 10 μg/μL, and crRNA and tracrRNA were suspended in RNase-free Duplex Buffer to a concentration of 200 μM. To prepare a well-structured RNA complex, equal volumes of crRNA and tracrRNA were combined and heated at 94 °C for 2 min and then placed at room temperature for 10 min. This annealed crRNA and tracrRNA complex was further mixed with Cas9 protein and ssODN so that the final concentration was 50 μM of crRNA/tracrRNA, 1 mg/mL of Cas9 protein, and 1 μg/μL of ssODN. Fast Green (Nacalai) was also added to the mixture at the final concentration of 0.02%. This mixture was diluted with Opti-MEM (Thermo Fisher Scientific) to adjust the volume to 1.5 to 2 μl/oviduct and then used for electroporation.

### Improved-Genome editing via Oviductal Nucleic Acids Delivery

To introduce amino acid substitution into Per1 or Per2 in *Per2^Luc^* C57BL6 mice, a genome editing method called *i*-GONAD was performed according to the protocol previously reported (34–36). Briefly, female mice in estrus were mated with stud male mice around 16:00. The onset of pregnancy was expediently defined at 0:00 on day 0. Female mice with copulation plugs were designated at day 0.4 of gestation (at 10:00) and anesthetized with isoflurane to perform electroporation under a dissecting microscope at day 0.7 of gestation (at 16:00), the time point corresponding to late one-cell stage of mouse embryogenesis, The ovary–oviduct–uterus complex was exposed outside the body after making an incision at the dorsal skin. Approximately 1.5 to 2 μl of electroporation solution as prepared above was injected into the oviduct lumen from upstream of the ampulla using an ultrafine glass micropipette. Immediately after the injection of solution, the oviduct regions were completely covered with a piece of Kimwipe (Jujo-Kimberly) well soaked in phosphate-buffered saline and then grasped with tweezer-type electrodes. Electroporation was performed using a square-wave pulse generator CUY21EDIT II (BEX). See detailed electroporation parameters in the original paper (34, 35). After electroporation, the ovary–oviduct–uterus complex was carefully returned to their original position, and the incision was sutured and treated with antibiotics. The animals were warmed until recovery from anesthesia. To confirm successful genome editing, genotyping of F0 generation was conducted using genomic DNA from mouse tail tissue. Briefly, DNA was extracted using the hot-alkaline method and used for PCR-based selection. PCR primers used are listed in Table S2. PCR products were digested with a specific restriction enzyme to distinguish whether each product was originated from a wild-type or mutant allele. Selected mosaic mice were back-crossed with *Per2^Luc^* C57BL6 mice and further crossed with littermates to generate mice that are homozygous for the mutant allele.

### Locomotor activity

Male mice aged 8 to 16 weeks were singly kept in standard mouse cages (W 213, D 324, and H 131 mm) placed in compartments equipped with infrared sensors to detect locomotor activity. Mice were allowed ad libitum access to food and water, which were changed every week. After habituation to the environment inside a compartment, the animals were well entrained on a 12:12-h LD cycle for 2 weeks and then exposed to 6-h phase-advanced LD cycles for 3 weeks. Subsequently, a constant darkness condition was started and continued for more than 6 weeks. Locomotor activity counts were obtained in real time using Clock Lab software (Actimetrics). The free-running circadian period length was calculated by χ^2^ method based on counts from the last 3 weeks of the experiment using the software ActogramJ provided by Dr Yoshii (44). To calculate the 50% phase shift value (PS^50^) for statistical evaluation of entrainment rate, a sigmoidal dose–response curve with Hill slope was fitted to onset time points using a least squares method. In these experiments, only male mice were used to monitor locomotor activity because the estrus cycle of female mice influences circadian rhythm in a measurable manner.

### Ex vivo tissue culture

Per2^Luc^-derived bioluminescence was monitored in tissue slice cultures as described previously (45). Briefly, Per2^Luc^ knock-in C57BL6 mice were euthanized with isoflurane and used to make 300-μm-thick slices from the SCN, liver, and lung with a McILWAIN tissue chopper, and the aorta was prepared with a stainless steel surgical blade in ice-cold Hank's balanced salt solution with 10 mM HEPES. Tissue explants on Millicell (Merck Millipore) were kept in Dulbecco's modified Eagle's medium with 0.035% sodium bicarbonate, 10 mM HEPES, 4.5 g/L D-glucose, 1.0% penicillin–streptomycin, and 10% FBS. Circadian rhythms of tissue slices were synchronized by incubating in DMEM containing dexamethasone at the final concentration of 100 nM for 1 h. Slices were then washed, and the medium was replaced with the same medium supplemented with 0.1 mM luciferin (Promega). Per2^Luc^-derived bioluminescence was measured in real time using an LM2400 (Hamamatsu Photonics) located inside an incubator. Period length, amplitude, and damping rate were calculated by fitting a model equation to the detrended data using the least squares method as described previously (46). The detrended datasets were obtained by subtracting the 24-h simple moving average from the raw data.

### Measurement of half-life of Per2^Luc^

To examine Per2 half-life under an ex vivo condition, the protein synthesis inhibitor CHX was directly added to culture medium in each culture dish at the final concentration of 100 μM, at the first peak of circadian rhythms of bioluminescence emitted from ex vivo cultured tissues. The decline in bioluminescence was monitored in real time using an LM2400. Half-lives were mathematically estimated by fitting decline data obtained from 1 to 24 h after addition of CHX to an exponential decay model curve as described previously (46).

### Quantification and statistical analysis

In general, data are represented as the mean ± SD. Specific statistical tests used, *P-*value level definitions, and additional details are described in each figure legend.

## Supplementary Material

pgae516_Supplementary_Data

## Data Availability

The data supporting the findings of this article are available within the article and/or its Supplementary material.
