# Supplementary material for: In vivo functional significance of direct physical interaction between Period and Cryptochrome in mammalian circadian rhythm generation
Source: PNAS Nexus. 2024 Nov 15;3(12):pgae516. doi: 10.1093/pnasnexus/pgae516 (PMC11645128; doi:10.1093/pnasnexus/pgae516)
Supplement: pgae516_Supplementary_Data [file pgae516_supplementary_data.zip › PNASNEXUS-PNASNEXUS-2023-01104RR-s01.pdf]

Per1$^{CACA/CACA}$ mice

crRNA:
CACAGTCCACACACGCCTTAGGG (3’)

ssODN:
TAGCAGGTTCCCAGTGAGGACACTACATAACTCATCCTTTCTTCTCTCCCTAAGGCGGCCGTCG
ACGCCGGCAGCAGCGTTCAAGATCCTGGCCACTCTGATGACCCCCTCTTCTCAGAA (3’)

Per2$^{CACA/CACA}$ mice

crRNA:
CTGTGTTTACTGCGAGAGTGAGG (3’)

ssODN:
AGATTCCAGCTGCTTGCTTCTTACTCATGTGACCTGTTCTCTCTGAAGGGCGCCGTATACGCCG
AAAGTGAGGAGAAAGGCAACATTTGCCTGCCATATGAGGAAGACAGTCCTTCCCCG (3’)

Per2$^{PT/PT}$ mice

crRNA:
AAGTGTAACAGTTACCGGGAGGG (3’)

ssODN:
CCCATCTGGCTGCTGATGGCCAACACAGACGACAGCATCATGATGACATACCAGCTAACGTCG
CGGTAACTGTTACACTTCCTGGGAAGCCTGGAGGGCTGGGTAGGCGTGTCCAACACC (3’)

Table S1 Sequences of crRNAs and ssODNs for i-GONAD
