# Supplementary material for: In vivo functional significance of direct physical interaction between Period and Cryptochrome in mammalian circadian rhythm generation
Source: PNAS Nexus. 2024 Nov 15;3(12):pgae516. doi: 10.1093/pnasnexus/pgae516 (PMC11645128; doi:10.1093/pnasnexus/pgae516)
Supplement: pgae516_Supplementary_Data [file pgae516_supplementary_data.zip › PNASNEXUS-PNASNEXUS-2023-01104RR-s02.pdf]

Per1$^{CACA/CACA}$ mice

Sense: (5’) AACCACATCACCTCGCAAGT
Antisense: (5’) GCTTGAACCCTTAGCCCCAA
Restriction enzyme recognizing the mutant allele: SalI

Per2$^{CACA/CACA}$ mice

Sense: (5’) ACCTGAGCATGTGCCATGAA
Antisense: (5’) AGGAACCAGGGCGTATCTCT
Restriction enzyme recognizing the mutant allele: Bst1107I

Per2$^{PT/PT}$ mice

Sense: (5’) ACGATCGGCGTTTCGTGTAT
Antisense: (5’) TGTAACACAGTTCAAGGGGCA
Restriction enzyme recognizing the wild-type allele: PvuII

Table S2 Sequences of genotyping primers
